# Occurrence of Autoimmune Diseases Related to the Vaccine against Yellow Fever

**DOI:** 10.1155/2014/473170

**Published:** 2014-10-22

**Authors:** Ana Cristina Vanderley Oliveira, Licia Maria Henrique da Mota, Leopoldo Luiz dos Santos-Neto, Jozélio Freire De Carvalho, Iramaya Rodrigues Caldas, Olindo Assis Martins Filho, Pedro Luis Tauil

**Affiliations:** ^1^Faculdade de Medicina, Universidade de Brasília, Brasília, DF, Brazil; ^2^CRDF, SHLN Bloco L Centro Cínico Norte II, 70910-900 Brasília, DF, Brazil; ^3^Hospital Universitário Professor Edgard Santos da Universidade Federal da Bahia, 70390-700 Salvador, BA, Brazil; ^4^Coordenação de Pesquisa da Fundação Oswaldo Cruz (FIOCRUZ), 41810-080 Brasília, DF, Brazil; ^5^Laboratório de Biomarcadores de Diagnóstico e Monitoração do Centro de Pesquisas René Rachou, FIOCRUZ, 21040-900 Belo Horizonte, MG, Brazil

## Abstract

Yellow fever is an infectious disease, endemic in South America and Africa. This is a potentially serious illness, with lethality between 5 and 40% of cases. The most effective preventive vaccine is constituted by the attenuated virus strain 17D, developed in 1937. It is considered safe and effective, conferring protection in more than 90% in 10 years. Adverse effects are known as mild reactions (allergies, transaminases transient elevation, fever, headache) and severe (visceral and neurotropic disease related to vaccine). However, little is known about its potential to induce autoimmune responses. This systematic review aims to identify the occurrence of autoinflammatory diseases related to 17D vaccine administration. Six studies were identified describing 13 possible cases. The diseases were Guillain-Barré syndrome, multiple sclerosis, multiple points evanescent syndrome, acute disseminated encephalomyelitis, autoimmune hepatitis, and Kawasaki disease. The data suggest that 17D vaccination may play a role in the mechanism of loss of self-tolerance.

## 1. Introduction

Yellow fever is an infectious disease endemic in South America and Africa. The presence of viral reservoirs and the large number of viral vectors are prerequisites for the outbreaks of yellow fever observed in these areas [1]. The virus is transmitted by the bite of blood-sucking insects of the family Culicidae, in particular the genera* Aedes* and* Haemagogus* [2].

The yellow fever virus is a single-stranded RNA virus that belongs to the family Flaviviridae [1, 2]. The virus most likely originated in Africa and was brought to America during the slave trade [1, 3].

Epidemiologically, yellow fever exists in two forms, rural and urban, which are similar in their clinical and pathophysiological characteristics [2]. The disease lethality is estimated between 5 and 40%. In more severe cases, yellow fever evolves with sepsis, ictero-, and hepatorenal hemorrhagic syndromes, myocardial injury, and shock [1, 2]. According to the World Health Organization (WHO), more than 50% of people without treatment will die from the disease [4]. Some series report 94,8% of lethality at severe disease [5].

The clinical manifestations range from oligosymptomatic forms to severe and fatal cases [1, 2]. Only one in seven infected individuals develops symptoms [6]. The extremes of age are related to the severity and lethality of infection [7]. The incubation period is 3–6 days [8]. In the milder forms, infected individuals may develop nonspecific malaise, myalgia, nausea and vomiting, fever, and headaches. In this presentation, severe hemorrhage, hematemesis, epistaxis, ecchymosis, jaundice, dehydration, and oligo/anuria complete the picture [1, 2, 7]. Infection with the yellow fever virus is distinguished from other flaviviruses by liver damage and jaundice. Late manifestations, such as mental confusion, seizures, and coma, can occur. Death occurs between 7 and 10 days after the onset of symptoms [1].

The best method to prevent yellow fever is vaccination [2, 8]. The 17D vaccine, made with live attenuated virus, has been used since 1937 and is considered one of the safest and most effective vaccines ever produced [2, 9]. The vaccine induces neutralizing antibodies in 90% of recipients after 10 days and in 99% of recipients after 30 days [6]. Today, the vaccines for yellow fever are derived from two substrains, 17D and 17D-204 [8]. The vaccines are produced by four centers: Institut Pasteur in Dakar, Senegal; Bio-Manguinhos Wire Cruz, Rio de Janeiro, Brazil; Sanofi Pasteur, US; and Sanofi Pasteur, France [1]. The WHO recommended that the vaccine be administered every 10 years until 2013. However, some authors believe that the vaccine's protection can extend throughout an individual's lifetime [2, 3]. Thus, based on the existing data, the WHO changed the recommendation: there is no need for revaccination for the immune-competent individuals [10]. It is estimated that over 500 million doses have been applied [9].

However, the yellow fever vaccine is not free of adverse effects [2, 8]. In general the adverse effects are mild and well tolerated, including pain in the injection site, headache, myalgia, fever, and lower back pain. These symptoms occur between the second and 11th day after the application [8]. Among the serious adverse events, a multisystem disease (YEL-AVD) and a neurological disease (YEL-AND) are associated with the yellow fever vaccination. Both are a consequence of infection by the vaccine virus [2, 8, 9]. YEL-AVD exhibits a clinical picture similar to wild yellow fever, whereas YEL-AND encompasses neurological manifestations, such as encephalitis and meningitis. Some autoimmune manifestations, such as Guillain-Barré or ADEM (acute disseminated encephalomyelitis), are also described [8, 11].

The development of autoimmune manifestations has been linked to vaccination and to infection by wild-type microorganisms [12]. Infections are potentially relevant events in the development of autoimmune diseases, as they have the ability to hyperstimulate the immune system. Antigens similar to human cells and tissues may stimulate autoantibody production because of the molecular mimicry in genetically predisposed individuals [9].

As the infection mechanisms due to the vaccine virus and wild microorganisms are the same, it is theoretically possible for autoimmunity to occur after vaccination [12, 13]. Another factor to be considered is the potential induction of autoimmunity by vaccine adjuvants [13].

In this study, we performed the first systematic review to identify the occurrence of autoimmune diseases related to the vaccine against yellow fever.

## 2. Methods

The search for articles was performed on September 9, 2012, in the following databases: PubMed, MEDLINE, LILACS, and SCiELO.

We used the words or phrases “yellow fever vaccine” or “yellow fever vaccination” in combination with “autoimmune,” “autoimmunity,” “immune response,” “adverse effects,” and “adverse events.”

We included only studies published between 1937, the year the 17D vaccine was introduced, and September 9, 2012. We limited the included studies to those performed on humans and on the 17D vaccine (17D, 17DD, or 17D 204). The adverse event must have occurred within 30 days of the vaccination. Only articles written in English, Portuguese, or Spanish were considered.

After the initial screening, duplicate articles and those which were not case series were excluded.

The remaining abstracts were read, and those that cited the occurrence of some autoimmune disease were selected for reading. For purposes of this review, autoimmune diseases are considered to be any autoimmune disorders that are characterized by the production of autoantibodies. These must react with host tissues or immune effector cells that are reactive to endogenous peptides.

We found 602 articles. After applying the inclusion criteria, 463 articles remained and were screened by title. Of those remaining, we selected case series and case reports, totaling 132 articles, of which 82 were duplicates. Thus, 50 articles were selected for an analysis of the abstracts. Selected articles should cite at least one occurrence of autoimmune disease related to the 17D vaccine. At the end of our selection, we were left with 6 articles.

The quality of the selected articles was assessed by the McHarm tool, developed by McMaster University, Canada [14].

## 3. Results

There were 13 cases time-related to the development of autoimmune diseases (Table 1). Eight men (61.5%) and 5 women (38.5%) comprised the sample. Their ages ranged between 12 and 68 years, with a mean age of 42.31 years and a standard deviation of 20.43 years. The hepatitis A vaccine was applied in 8 of the cases described in this work. The time between the application of the vaccine and the clinical manifestations ranged between 7 and 27 days, with an average of 13.92 days.

In 1967, Miller et al. reported a series of 9 cases related to vaccination and multiple sclerosis. One case concerns a woman, aged 22 years, vaccinated against yellow fever in 1942. Six weeks prior to her yellow fever vaccine, she had been immunized with tetanus, typhoid vaccine, and smallpox without adverse effects. Hours after receiving the vaccine against yellow fever, she developed progressive loss of vision in both eyes, with recovery in a few weeks. One year after receiving the yellow fever vaccine, retrobulbar neuritis presented in the right eye. Over 12 episodes of retrobulbar neuritis between 1944 and 1951 were reported by the authors. The patient exhibited unilateral or bilateral paresis and emotional lability. Fifteen years after the vaccination, an examination revealed sequelae such as atrophy of the left optic nerve, residual intention tremor, and ataxia. The remaining cases cited by Miller involved vaccinations against other diseases [15].

In 2009, Schmöeller and colleagues described an atypical case after yellow fever vaccination that occurred in Rio Grande do Sul, Brazil. A male child, aged 12 years, developed the typical manifestations of Kawasaki disease. Kawasaki disease is a vasculitis of medium-caliber vessels with involvement of cardiac vessels and possible autoimmune etiology. Twenty days after the vaccination, the patient developed fever, fatigue, and myalgia, followed by conjunctivitis and cervical lymphadenopathy. Investigations revealed leukocytosis (17 × 10^9^ cells/L), eosinophilia (1.5 × 10^9^ cells/L), and moderate anemia. The erythrocyte sedimentation rate was 112 mm/h. Tests for cytomegalovirus, Epstein-Barr virus, and toxoplasmosis were negative. Echocardiography revealed coronary dilation. Treatment was started after the diagnosis, and the patient was medicated with immunoglobulin (2 g/kg, one day) plus aspirin. There was improvement for 2 weeks followed by arthralgia and recrudescence of conjunctivitis. The team opted to start prednisone therapy at 10 mg/kg, and symptom remission was observed. One month later, the child exhibited improved echocardiographic parameters and good recovery. The child remained asymptomatic and continued low-dose-aspirin therapy according to the authors of the publication [17].

Stangos and colleagues described a case of multiple evanescent white dot syndrome (MEWDS) after simultaneous vaccination against hepatitis A and yellow fever. This disease is benign, is characterized by white spots in the retinal pigment epithelium, and manifests clinically by unilateral involvement with painless loss of visual field scotomas and fotopsias. The disease mainly affects females, as in the case described. This patient was a woman of 50 years who developed visual loss in photopsia and painless paracentral scotoma in the left eye 10 days after vaccination. Examination revealed a visual acuity of 20/40, and fundoscopy revealed papillitis, macular granularity, and multiple white spots. Fluorescein angiography of the affected eye revealed numerous hypofluorescent injuries gradually radiating from the optic nerve. These findings, associated with the exclusion of sarcoidosis, tuberculosis, and syphilis, led researchers to diagnose MEWDS. The patient exhibited spontaneous clinical remission after 6 weeks, with normalization of her examinations. The authors believe that her older age might have favored the development of MEWDS [18].

Vital et al. analyzed the case of a 66-year-old patient vaccinated against yellow fever who developed progressive motor and sensory neuropathy associated with bilateral facial paralysis 15 days after vaccination. CSF analysis revealed a protein concentration of 3 g/L and 5 lymphocytes. Six weeks after the onset of symptoms, the patient underwent a tracheotomy. The patient required rehabilitation for 9 months. In the paper, there is no detailed description of the type of rehabilitation or the drugs that were used. Biopsy samples were necessary and were taken from the superficial peroneal nerve and peroneal muscle. Histological analysis revealed destruction of the myelin sheath and associated macrophages [19].

There are also reports of a 31-year-old patient, previously healthy, who developed autoimmune hepatitis 11 days after vaccination against hepatitis A and yellow fever. Perumalswami and colleagues described the clinical findings, which began with fever, dark urine, nausea, and vomiting. On physical examination, the patient exhibited jaundice and hepatomegaly. Laboratory tests reported values of 12 g/dL hemoglobin, 575 U/L ALT, 1276 U/L AST, and 162 U/L alkaline phosphatase. Serology for Epstein-Barr virus, leptospira, and hepatitides A, B, C, and E were negative. The following autoantibodies were measured and showed no reactions: anti-nuclear antibodies (ANA), liver-kidney microsomal (LKM) antibody, antimitochondrial antibodies, and anti-smooth muscle antibodies. A slight increase in IgG that reached 18.7 g/L was observed (VR < 17.28 g/L). PCR for viral RNA of the yellow fever virus was negative. The patient's clinical and laboratory findings improved over 3 weeks. New analysis indicated further increases in transaminases and bilirubin. The presumptive diagnosis of autoimmune hepatitis was made based on the criteria of the International Autoimmune Hepatitis Group (International Autoimmune Hepatitis Group (IAIGH)). Liver biopsy revealed lymphoplasmocytes periportal infiltrates, parenchymal necrosis, and lobular inflammation. The patient was treated with 40 mg of prednisone and 50 mg of azathioprine per day. After 3 months, the transaminases had normalized. After 1 year, a biopsy indicated a significant reduction in periportal inflammation. Previously necrotic areas were replaced by fibrous septa [16].

By analyzing data from 1990 in the 2005 American surveillance system, “Vaccine Adverse Event Reporting System (VAERS),” McMahon and colleagues sought to identify neurological syndromes associated with vaccination against yellow fever. Acute disseminated encephalomyelitis (ADEM) is defined by the presence of disseminated demyelination on imaging, as supported by the clinical presentation. Guillain-Barré syndrome (GBS) is defined by the presence of peripheral neuropathy and electrodiagnostic findings consistent with acute axonal demyelination. For the purpose of their analysis, encephalitis had to be caused after vaccination. Therefore, it is necessary to confirm the presence of the virus or specific IgM in the CSF. GBS and ADEM are considered autoimmune events. The authors considered a range of 30 days to classify the events as related to the yellow fever vaccine. We identified 3 cases of suspected ADEM in patients between 18 and 61 years of age. The clinical symptoms began between 7 and 20 days after vaccination. One of these patients exhibited retrobulbar optic neuritis with bilateral eye pain, blurred vision, numbness in the upper limb, lower limb paresis, and difficulty urinating. The patient developed persistent paresthesia but with almost total recovery of the other symptoms. The others, aged 19 and 61 years, progressed to paresis and difficulty walking. The oldest did not fully recover and was later referred to a recovery center. The youngest, although he exhibited associated urinary dysfunction, showed improvement and was able to walk without assistance. This last patient, diagnosed with ADEM, was not considered by our criteria to exhibit positive CSF IgM, indicating that the vaccine virus did not penetrate into the central nervous system. This consideration was not made by the authors. Six patients, aged 17 to 68 years, developed suspected Guillain-Barré between 7 and 27 days after immunization. IgM CSF was evaluated in only one case and produced a negative result. Three patients required immunoglobulin, one patient required plasmapheresis, and another patient required two high doses of metilprednisolone. Of the six patients, one had diplopia at discharge, whereas the others exhibited minimal sequelae. None of the patients diagnosed with Guillain-Barré or ADEM in this publication had received the yellow fever vaccine alone [11].

Although it has been retracted because the author of the revision considered the disease secondary to an infection with the vaccine virus, Merlo et al. describe one possible case in 1993. The author published the first time-related description of vaccine associated encephalitis due to 17D in an adult. The patient was a 29-year-old individual immunized for the first time against yellow fever. The patient received vaccinations against diphtheria and tetanus on the same day as the yellow fever vaccine. At the third day, the patient had fever and headache and vomited. Nineteen days after, his clinical findings consisted of blurred vision, headache, dizziness, and ataxia. The sequela was persistent mild ataxia. Serological investigation for neurotropic viruses and bacteria was negative. The yellow fever neutralizing antibody was found in serum after 60 days (titer 1 : 80). Specific IgM or viral RNA in the CSF were not measured, and, thus, it was not possible to determine whether the symptoms were due to infection with the vaccine virus or to the occurrence of a secondary autoimmune response [20].

## 4. Discussion

Several studies in the literature have reported the association of vaccination with the development of autoimmune diseases [12]. Autoimmune events after vaccination are rare; therefore, the number of subjects studied would need to be quite high to identify the appropriate number of cases. Accordingly, the human and material cost becomes high in cohort studies. Additionally, it is difficult to control all of the factors that can alter the results of the study, such as environmental factors, genetic factors, or infections. The unpredictability of autoimmunity and the lack of previous studies make the study design even more complex. Even though one might identify the presence of an eventual relationship between the 17D vaccine and autoimmunity, new studies are needed to establish a relation cause and effect.

Although there are few case reports, a series of cases presents other limitations. The identification of cases was conducted passively, leading to underreporting. The amount of information collected and the data quality may be different in each study. Some of the reasons are the lack of resources, delayed diagnosis, or differences between examiners. Although the vaccines are derived from the same original strain, they are produced by four distinct centers, and differences in handling and preservatives may occur. Such differences can be responsible for the development of autoimmune events. Thus, the comparison between cases involving vaccines of different manufacturers can lead to less reliable results. However, given the lack of studies on the topic, a series of cases remains an important tool.

Our study was heavily weighted by McMahon data, which were extracted from VAERS. It is a system of passive reporting and it was not designed for the establishment of cause and effect relationship. Some of its limitations are variability of data quality, underreporting, lacking of denominator information, and reporter bias. However, this system identifies adverse effects related to vaccines that must be further studied. Since there is a little amount of scientific literatures to establish or refute causal relationships for many adverse effects, VAERS is an important tool to be considered because it may highlight the red flags about vaccines [21].

Several MS cases have been reported around the time of vaccination, notably with hepatitis B [12, 13, 22]. The possible mechanisms involved include molecular mimicry between proteins of hepatitis B virus and myelin, the general immune stimulation by antigens or even toxic contaminants of the vaccine [22]. Among the myelin antigens, the major target of multiple sclerosis (MS) and experimental autoimmune encephalomyelitis (EAE) is the myelin basic protein (MBP). The humoral immunity against myelin oligodendrocyte glycoprotein (MOG) is related to myelin damage. The MBP and MOG share homologies with the antigen of hepatitis B, HBsAg. Using synthetized peptides that mimic MBP and MOG, Bogdanos et al. studied 234 serum samples and identified double reactivity between HBsAg and MOG after hepatitis B immunization. At 3 months postvaccination reactivity to at least one of the MOG mimics was present in 53% of the cases. The data suggest that HBsAg share important homologies with myelin antigens and that the antiviral response can induce cross-reactive anti-self immune responses that reduce after time. The author indicates that the tolerance mechanisms may be responsible for this decrease [23]. The number of cases reported following immunization against yellow fever is considerably lower compared to hepatitis B. However, among autoimmune events reported following yellow fever vaccine, myelin is the target in more than 80% of the cases described. Therefore, it is plausible that the mechanisms suggested for hepatitis B may also apply to the yellow fever vaccine.

In evaluating the studies, some limitations should be considered. Some studies have reported the specific type of yellow fever vaccine. However, since 1937, the 17D vaccine has been used. This can be derived from two different 17D substrains, DD-17 and D-17 204, which differ by the number of passages in murine tissues and chickens [8]. Despite the existence of two subtypes of the vaccine in addition to the original 17D vaccine, the absence of this information does not invalidate the studies presented.

The type of surveillance used in our study, often passive, underestimates the actual number of cases. Furthermore, different countries have different ways of collecting data and different methods of diagnosis, which may affect the outcome. In some cases included in this series of cases, there was no detailed information on the diagnostic procedures performed or the possible exclusion of other differential diagnoses.

The time between the onset of symptoms and the immunization can also interfere with the data analysis. Most of the studies regarding adverse events related to yellow fever vaccine consider a 30-day period for analysis. Autoimmune events may occur over periods longer than 30 days and are not considered related to the vaccine. The development of autoimmune events and autoimmune diseases is rare. Because they are nonspecific, they can be very difficult to be diagnosed. Thus, it is possible that there is a subnotification. On the other hand, symptoms caused by other variables, such as other virus infections or bad alimentation, must be interpreted as 17D adverse events if they occur within 30 days.

Many studies included in this series of cases describe adverse events after administering more than one vaccine at a time. In this situation, it is not possible to attribute the adverse effects solely to the vaccine against yellow fever. The case of multiple evanescent white dot syndrome (MEWDS) described by Stangos et al. occurred after simultaneous vaccination against hepatitis A and yellow fever [18]. However, a case of MEWDS has been reported by Fine in a male patient of 30 years, 13 days before vaccination against hepatitis A alone [24]. Thus, there is no way to exclude the participation of any of the vaccines. The disease may have developed by chance or was caused by one or both vaccines. Perumalswami and colleagues also describe autoimmune hepatitis in this case after simultaneous vaccination against hepatitis A and yellow fever [16].

There is no description of synergism between vaccines in the induction or promotion of self-reactive events. Vaccination against multiple agents has been reported to be well tolerated but should be avoided in those more susceptible to autoimmune events (e.g., those with a family history or previous occurrence of other autoimmune diseases) [16].

Exacerbation of preexisting autoimmune diseases was not identified in this series of cases. Our group identified 70 patients with rheumatic diseases who were inadvertently vaccinated against yellow fever [25]. In this study, only mild adverse effects were observed (mild skin rash, headache, myalgia, and transient elevation of liver enzymes) at a frequency similar to that reported in the population without a diagnosis of autoimmune diseases. Among patients evaluated by Mota et al., there were cases of rheumatoid arthritis, systemic lupus erythematosus, systemic sclerosis, and spondyloarthritis, and no cases of reactivation or flare of the underlying disease in the immediate period after the yellow fever vaccination were observed. The lack of studies evaluating the activity of autoimmune diseases is due to the inability to perform controlled studies due to increased risk of YEL-AVD in this group of individuals. Because the vaccine is made from the live virus, it is contraindicated in immunocompromised individuals [8, 9, 26].

The use of adjuvants in vaccines was also related to the appearance of autoantibodies and constitutional symptoms [27, 28]. The antiphospholipid syndrome can be induced by tetanus vaccine in murines [29]. Anticardiolipin autoantibodies can be elevated after influenza immunization in patients with lupus [30]. The adjuvant's use in the manufacture of vaccines increases the immune response and its duration against the antigen, promoting physical protection against the pathogen and facilitating its translocation to lymph nodes [27]. In 2001, Schoenfeld and Agmon-Levin described a syndrome known as autoimmune/inflammatory syndrome induced by adjuvants (ASIA). This syndrome includes 4 conditions with common features and exposure to a component with adjuvant effect: macrophagic myofasciitis syndrome, Gulf War syndrome, Siliconosis, and the adjuvant disease. Some criteria were suggested. The major criteria are exposure to an external stimuli, typical clinical manifestations (myalgia, myositis, muscle weakness, arthralgia/arthritis, chronic fatigue, sleep disturbances, neurological manifestations, cognitive impairment, memory loss, and pyrexia), improvement while removing the agent, and typical biopsy. The minor criteria are the appearance of antibodies or autoantibodies related to the suspected adjuvant, other clinical manifestations, specific HLA, and evolvement of autoimmune disease [27]. The syndrome is very rare although the exposure to antigens is very common, which suggests other risk factors such as genetic features or coexposure [31].

Although the 17D yellow fever vaccine has its limitations, such as the risk for immunocompromised persons, it is the safest and most effective arboviral vaccine [32]. The development of an inactivated vaccine would be a solution for the immunosuppressed ones. The virus inactivation made by pressure could reduce the use of external agents. Thus, the yellow fever vaccine could be more secure [32]. However, killed vaccines may have disadvantages as risk of incomplete inactivation, change in immunogenic properties of the virus, and the use of multiple doses [32, 33].

Among the abstracts selected for reading, there was greater interest in the occurrence of YEL-AVD; 27 cases were identified in all. It is believed that the occurrence of serious adverse effects is related to the host. Therefore, it is possible that autoimmune phenomena may be related to host characteristics as well [34, 35].

## 5. Conclusion

Although we have to consider the limitations of the data presented, due to the scarcity of information on the subject and quality of available studies, this is the first systematic review to evaluate events specifically related to autoimmunity developing after vaccination against yellow fever.

While it is plausible to consider the possibility of autoimmune events after yellow fever vaccine, considering the massive coverage in endemic regions, a systematic review of the literature reveals that there are few reports of such autoimmunity. The data set found may suggest that the increase in risk of autoimmunity when the yellow fever vaccine is applied is unlikely.

## Figures and Tables

**Table 1:** Cases of autoimmune diseases after yellow fever vaccination.

	Author	Disease	Age (years)	Gender	Time between vaccine and symptoms (days)	Symptoms	McHarm (quality)	Other vaccines
1	McMahon et al. [11]	GBS	49	M	10	Headache, diplopia	Good	Meningitis, dT, typhoid fever, Salk
2	McMahon et al. [11]	GBS	37	M	16	Headache, blurred vision	Good	Hep B, meningitis, dT
3	McMahon et al. [11]	GBS	17	F	7	Difficulty breathing	Good	Typhoid, Hep A, dT
4	McMahon et al. [11]	GBS	56	M	27	Paresis	Good	Hep A, Hep B, typhoid fever, meningitis
5	McMahon et al. [11]	GBS	63	F	16	Headache, unsteady gait	Good	Hep A
6	McMahon et al. [11]	GBS	68	F	8	Paresis	Good	dT, Hep A, typhoid
8	McMahon et al. [11]	ADEM	18	M	20	Eye pain, paresis, blurred vision	Good	dT, MMR
9	McMahon et al. [11]	ADEM	61	M	7	Unsteady gait	Good	dT, hepatitis A
10	Miller et al. [15]	MS	22	M	“Few hours”	Visual loss, paresis	Bad	Typhoid, tetanus, chickenpox
11	Perumalswami et al. [16]	AIH	31	F	11	Liver failure	Intermediate	Hep A
12	Schmöeller et al. [17]	KD	12	M	20	Conjunctivitis, fever, coronary aneurism	Bad	None
13	Stangos et al. [18]	MEWDS	50	F	10	Temporary visual loss	Intermediate	Hepatitis A
14	Vital et al. [19]	MS?	66	M	15	Facial paralysis	Bad	None

GBS: Guillain-Barré syndrome; ADMS: acute demyelinating multiple sclerosis; AIH: autoimmune hepatitis; KD: Kawasaki disease; MEWDS: multiple evanescent white dot syndrome; dT: dyphteria and tetanus; Hep B: hepatitis B; Hep A: hepatitis A; MMR: Measles-Mumps-Rubella.

## Abbreviations

WHO: World Health Organization
YEL-AVD: Yellow fever vaccine associated viscerotropic disease
YEL-AND: Yellow fever vaccine associated neurotropic disease
ADEM: Acute disseminated encephalomyelitis
MEWDS: Multiple evanescent white dot syndrome
GBS: Guillain-Barré syndrome.
